# Effect of *Trichoderma velutinum* and *Rhizoctonia solani* on the Metabolome of Bean Plants (*Phaseolus vulgaris* L.)

**DOI:** 10.3390/ijms20030549

**Published:** 2019-01-28

**Authors:** Sara Mayo-Prieto, Roberta Marra, Francesco Vinale, Álvaro Rodríguez-González, Sheridan L. Woo, Matteo Lorito, Santiago Gutiérrez, Pedro A. Casquero

**Affiliations:** 1 Grupo Universitario de Investigación en Ingeniería y Agricultura Sostenible (GUIIAS), Instituto de Medio Ambiente, Recursos Naturales y Biodiversidad, Universidad de León, Avenida Portugal 41, 24071 León, Spain; alrog@unileon.es (Á.R.-G.); pacasl@unileon.es (P.A.C.); 2 Dipartimento di Agraria, Università degli Studi di Napoli Federico II, Via Università 100, 80055 Portici (NA), Italy; robmarra@unina.it (R.M.); lorito@unina.it (M.L.); 3 Istituto per la Protezione Sostenibile delle Piante, Consiglio Nazionale delle Ricerche, Via Università 133, 80055 Portici (NA), Italy; francesco.vinale@ipsp.cnr.it (F.V.); woo@unina.it (S.L.W.); 4 Dipartimento di Farmacia, Università degli Studi di Napoli Federico II, Via Domenico Montesano, 49, 80131 Napoli, Italy; 5 Grupo Universitario de Investigación en Ingeniería y Agricultura Sostenible (GUIIAS), Área de Microbiología, Escuela de Ingeniería Agraria y Forestal, Universidad de León, Campus de Ponferrada, Avenida Astorga s/n, 24401 Ponferrada, Spain; s.gutierrez@unileon.es

**Keywords:** metabolomics analysis, bean, *Phaseolus vulgaris*, *Trichoderma*, *Rhizoctonia solani*, phytoalexins

## Abstract

The common bean (*Phaseolus vulgaris* L.) is one of the most important food legume crops worldwide that is affected by phytopathogenic fungi such as *Rhizoctonia solani*. Biological control represents an effective alternative method for the use of conventional synthetic chemical pesticides for crop protection. *Trichoderma* spp. have been successfully used in agriculture both to control fungal diseases and to promote plant growth. The response of the plant to the invasion of fungi activates defensive resistance responses by inducing the expression of genes and producing secondary metabolites. The purpose of this work was to analyze the changes in the bean metabolome that occur during its interaction with pathogenic (*R. solani*) and antagonistic (*T. velutinum*) fungi. In this work, 216 compounds were characterized by liquid chromatography mass spectrometry (LC-MS) analysis but only 36 were noted as significantly different in the interaction in comparison to control plants and they were tentatively characterized. These compounds were classified as: two amino acids, three peptides, one carbohydrate, one glycoside, one fatty acid, two lipids, 17 flavonoids, four phenols and four terpenes. This work is the first attempt to determine how the presence of *T. velutinum* and/or *R. solani* affect the defense response of bean plants using untargeted metabolomics analysis.

## 1. Introduction

The common bean (*Phaseolus vulgaris* L.) is one of the most important food legume crops worldwide. Bean cultivation can be affected by biotic and abiotic stresses, such as phytopathogen attack, drought, and cold [1]. Bean immunity, similarly to other plants, consists of the development of physical and biochemical barriers, plus the induction of defense responses. A passive resistance protection system, involving structural barriers and metabolites, prevents or attenuates invasion by potential attackers [2,3]. In addition, plants employ a number of chemical defenses consisting on secondary metabolites that act as signaling molecules, antimicrobials (i.e., phytoanticipins and phytoalexins), herbivore feeding deterrents and cell wall strengthening precursors [4]. However, certain plant pathogens can manipulate or overcome these chemical defenses, thereby establishing successful infections that would have a negative impact on plant growth and subsequent crop yield. To overcome this, the agricultural industry has relied on the use of agrochemicals such as pesticides and fungicides [3]. However, continuous application of such chemicals has shown negative impacts on the environment, creating a need for new environmentally-friendly strategies that fight against these diseases and pests by boosting the plants immune system [4].

*Rhizoctonia solani* JG Kühn [Teleomorph: *Thanatephorus cucumeris* (AB Frank) Donk] is a necrotrophic fungal pathogen, causing root and hypocotyl diseases. Rhizoctonia root rot of bean is common worldwide and it is one of the most devastating diseases of bean in large and small scale plantings [5].

Biological control represents an alternative to synthetic pesticides against phytopathogenic agents. Plant beneficial fungi of the genus *Trichoderma* (Teleomorph: *Hypocrea*) have been successfully used worldwide both to control fungal diseases and to promote plant growth. *Trichoderma* spp. represent a fundamental component of the rhizosphere microbiome because these fungi help plants to overcome numerous environmental constraints by stimulating defense responses and improving fitness and development [6]. These abilities have supported the application of *Trichoderma* strains as biocontrol agents (BCA) or plant biostimulants in agriculture and forestry. 

*Trichoderma* beneficial strains trigger the activation of plant cascade signals including the secretion of antimicrobial reactive oxygen species (ROS), the production of secondary metabolites such as phytoalexins and pathogenesis-related proteins, and the deposition of callose [7]. *Trichoderma* has evolved to interact with plants in such a way that it is not perceived as an enemy [6].

After the invasion by a pathogen or a biocontrol agent, the plant responds by inducing the expression of defense genes [8] and producing some bioactive secondary metabolites, [9] ranging from phytoalexins (phenols, isoflavones, terpenes) to substances that can block the invasion and spread of the pathogen, such as lignin and callose [10]. Biosynthesis of phytoalexins by plants in the Fabaceae family is a subject of interest because they are produced in higher or lower amounts depending on whether the plant is interacting with a pathogen or with a biocontrol agent.

Polyphenols, including phytoalexins, are widely distributed in plants and may be classified into three groups: (i) simple phenols, (ii) phenolic acids (hydroxycinnamic- and benzoic-acid derivatives), and (iii) flavonoids (e.g., flavones, flavanones, flavanonols, flavanols, isoflavones and lignans) [11]. Flavonoids and their derivatives comprise a large group of secondary metabolites whose production is specifically induced by symbionts and pathogens [12]. One of the roles of flavonoids inside the root could be the regulation of the defense response and it has been proposed that mycorrhiza invasion triggers a temporary defense response in the root, which involves induction of phytoalexins [13]. Isoflavonoids are a subclass of the flavonoids and they are thought to represent the majority of the phytoalexins produced by legume plants [12].

Other phytoalexins include toxins of terpenic nature, which act as repellents against many plant feeding-insects and -mammals, thus playing important plant defensive roles [14]. These compounds (i.e., triterpenes and saponins) have also been found in legumes and their activity as antimicrobial defense compounds has been reported [15].

In this work, we aimed to evaluate the changes in the bean metabolome that occur during the interaction with pathogenic (*R. solani*) and antagonistic (*T. velutinum*) fungi. The production of polyphenols and other metabolites by bean plants was analyzed by LC-MS analysis and differences in single or combined plant interacting metabolomes were investigated. 

## 2. Results

### 2.1. Analysis of Phaseolus vulgaris Plant Metabolome in Presence of T. velutinum and R. solani

*T. velutinum* T028 was selected based on its positive effect on bean plants growth. Previously, plants inoculated with this strain had shown a significant increase in dry weight of both the aerial part and root system, even when *R. solani* was present in the soil [8]. Thus, based on these results, that isolate was used in this study to further investigate its beneficial effect on plant metabolome.

**Figure 1:**
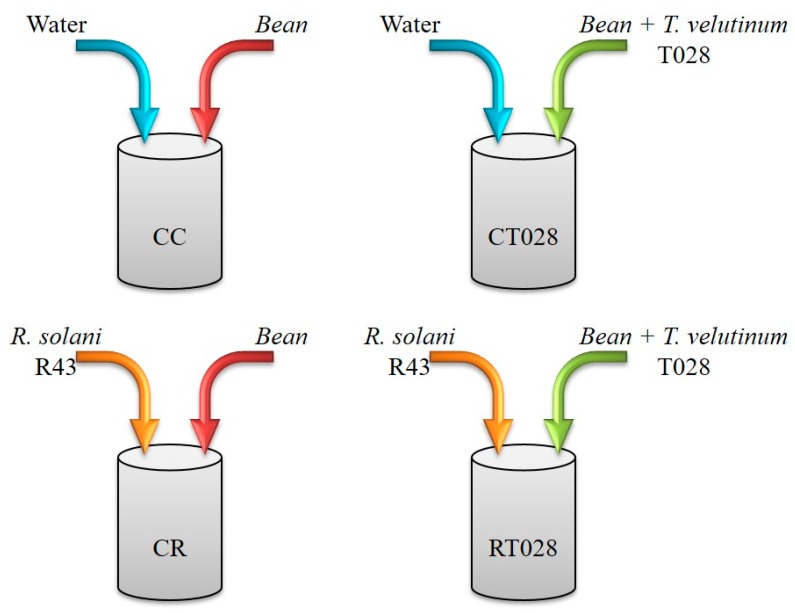
Scheme of the treatments performed in this work: CC, water control plants without fungal treatments; CT028, seeds coated with *T. velutinum* grown in uninfected soil; CR, untreated seed grown in *R. solani* (R43)-infected soil; and RT028, seeds coated with *T. velutinum* (T028) grown in *R. solani* (R43)-infected soil.

**Figure 2:**
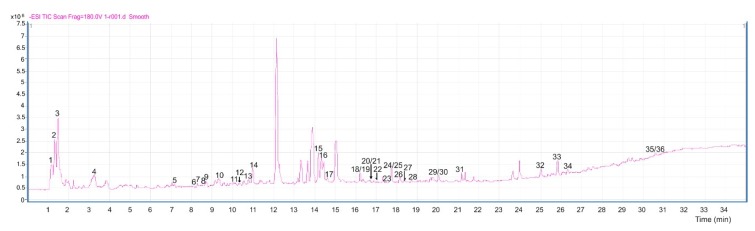
ESI-TIC chromatogram of leaf extracts obtained from control plants (CC), separated by LC-MS. See Table 1 for identification of the detected compounds.

HPLC analysis of bean leaf extracts revealed differences in the accumulation pattern of compounds, according to the interaction performed in vitro (see Figure 1). A total of 216 compounds were extracted from bean leaves and analyzed by LC-MS-qTOF in the samples. The use of optimal gradient elution programs and MS conditions on a negative ionization mode resulted in an ESI-TIC (Total Ion Current) chromatogram for each bean leaf sample (Figure 2). The compounds were tentatively identified by analyzing their mass spectra, determined via TOF-MS, and taking into account the data reported in the literature.

Metabolomic analysis of bean samples revealed up to 36 specific compounds associated with the Fabaceae family that accumulated differentially in the leaves of plants inoculated with the fungal isolates when compared to the controls (Table 1). The compounds from uninoculated bean plants (CC) were used as controls for the comparisons with two-way (CT028-CC and CR-CC) and three-way interactions (RT028-CC) (Figure 1). The compounds were considered as differentially produced when a 2-fold increase (UP) or decrease (DOWN) was observed compared to uninoculated plants (CC). Semi-quantification of each compound was performed by determining the area of their peaks on the chromatogram of each bean leaf extract and normalizing the data by calculating the relative ratio of abundance of metabolites in respect to all other peaks [16]. 

**Figure 3:**
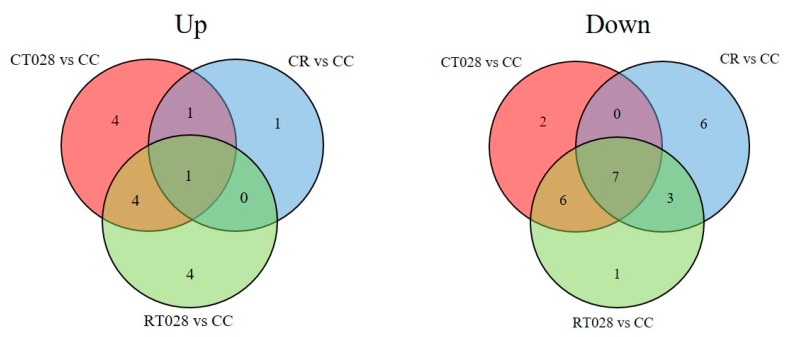
Venn diagram with the compounds with an Up-production and Down-production. See Table 2 for the putatively identified compounds in the interactions.

Several compounds were produced at significantly different levels in fungal-inoculated conditions compared to the control plants. The Venn diagrams reported in Figure 3 show the distribution of differential metabolites (UP or DOWN) accumulated in the plant metabolome during the interactions with *Trichoderma* alone (CT028), with the pathogen alone (CR) or with both fungi (RT028), compared to control, water-treated, plants (CC). Interestingly, some compounds were up- or down-produced in all the interactions examined (1 and 7, respectively, for UP and DOWN metabolites) but most of the differentially produced metabolites were found to be specifically influenced by the presence of one or both fungi. 

In all interactions including *T. velutinum*, bean leaves UP-produced 10 metabolites and DOWN-produced 15. Among these, four were exclusively UP-produced and two DOWN-produced, in interaction with the beneficial fungus (CT028) in all cases versus the control condition (CC) (Figure 3).

In the interaction with *R. solani* (CR), 19 compounds were differentially produced. These include three UP- and 16 DOWN-produced metabolites when compared to control plants (Figure 3).

In the three-way interaction, plant-*T. velutinum* -*R. solani* (RT028), 26 compounds were produced at significantly different levels compared to control plants. However, most of these metabolites were in common with the two-way interactions where only the beneficial or pathogen fungi was present (Figure 3).

### 2.2. Characterization of Compounds in Bean Leaf Metabolome

Table 2 includes the metabolites discriminating plant metabolomes obtained during single and combined interactions. These compounds were identified by LC-MS-qTOF analysis (operating in negative ion mode), by comparing the data with previously described compounds, which are present in both public and in-house databases (METLINE, PubChem, ChemSpider, HMDB, CAS, literature) including natural compounds and plant secondary metabolites. Putative metabolite identifications were performed using the “MassHunter Mass Profiler Software” (Agilent Technologies, Santa Clara, CA, USA) and selecting matches with high scores. The selected compounds were grouped in the categories listed below.

### 2.3. Amino Acids and Peptides

Statistical analysis revealed the different accumulation in the plant metabolome of several compounds that were tentatively identified as two amino acids and three peptides (Table 1). In particular, compound #8 [*m/z* 203.0826; retention time (RT) = 8.52 min] and compound #15 [(*m/z* 245.09315, RT 14.20 min], corresponding to the molecular formulas C_11_H_12_N_2_O_2_ and C_13_H_14_N_2_O_3_, respectively, were putatively identified as L-tryptophan and N-acetyltryptophan. Both metabolites were DOWN-produced in bean leaves when *R. solani* was present (CR) compared to the control (CC) (Table 2). However, the presence of *T. velutinum* increased their production both in two- and three-way interactions (CT028 and RT028) (Table 2).

Furthermore, three peptides (Table 1) were detected: Valyl-leucine [#7, *m/z* 229.1557, RT 8.42 min, (C_11_H_22_N_2_O_3_)] accumulated remarkably in presence of *T. velutinum* (CT028 and RT028) rather than in control plants but at a lower level in the sole presence of *R. solani* (CR) (Table 2); Ile-Gln-Ile [#11, *m/z* 371.2298, RT 10.00 min, (C_17_H_32_N_4_O_5_)], whose production was reduced by the presence of *R*. *solani* (CR and RT028); Gln-Gln-Gln [#29, *m/z* 401.1788, RT 19.94 min, (C_15_H_26_N_6_O_7_)] whose production decreased in presence of *T. velutinum* (CT028 and RT028) (Table 2).

### 2.4. Carbohydrates and Glycosides

The production of sucrose (Table 1) [#2, *m/z* of 341.1087, RT 1.43 min, (C_12_H_22_O_11_)] was detected in two-way interactions (CT028 and CR) but not when both fungi were together (RT028) (Table 2).

l-Ascorbic acid-2-glucoside (Table 1) was also found, [#4, *m/z* 337.0775, RT 4.96 min, (C_12_H_18_O_11_)], showing UP-production only in the presence of the pathogen, compared to the plant alone (Table 2*)*.

### 2.5. Fatty Acids and Lipids

Three compounds belonging to this group were found in bean metabolome (Table 1). These include γ-Linolenic acid [#35, C_18_H_30_O_2_], 13(S)-HOTrE [#34, C_18_H_30_O_3_], and PA(O-16:0/12:0) [#36, C_31_H_63_O_7_P]. Significantly lower amounts of all of them were produced compared to control plants (DOWN-produced) when the pathogen *R. solani* was present (CR and RT028) (Table 2). Moreover, 3-*O*-α-l-rhamnopyranosyl-3-hydroxydecanoic acid was also putatively identified [#30, C_16_H_30_O_7_], showing a significantly decreased amount (DOWN-produced) in plants inoculated with *Trichoderma* (both in CT028 and RT028), compared to control plants (CC) (Table 2).

### 2.6. Phenols

A huge number of phenolic compounds were detected (Table 1). These include: 

(i) Flavones. Compounds #22 and #27, with *m/z* 373.0926 and 389.0874, corresponding to the formula C_19_H_18_O_8_ and C_19_H_18_O_9,_ were homologues to 3′,5-dihydroxy-3,4′,6,7-tetramethoxyflavone [#22] and 5,2′,4′-trihydroxy-3,7,8,5′-tetramethoxyflavone [#27], respectively. Both compounds were UP-produced in plants inoculated with both fungi, either alone or combined (CT028, CR and RT028) (Table 2).

(ii) Flavonols. Quercetin 3-vicianoside [#12], *n*-octyl-β-d-maltopyranoside [#26] and sitosterol [#21] accumulated differently according to the interaction examined. Compounds #12 and #21 were more abundant in presence of *T. velutinum* (both in CT028 and in RT028) (Table 2). Conversely, compound #26 decreased in presence of this fungus (CT028).

(iii) Flavonones. Compound #3 showed remarkable homologies with naringin [*m/z* 579.1773, C_27_H_32_O_14_], a flavonone produced particularly in the sole presence of *T. velutinum* (CT028) (Table 2).

(iv) Flavonoid glycoconjugates. Numerous putative flavonoids conjugated with different polysaccharides were found in the bean metabolome during the interaction with *T. velutinum* and *R. solani*. These include glucopyranosides [#1, #10, #19, #24] and glucosides [#14, #16, #18, #20] (Table 2). The relative abundance of such compounds varied between treatments: Compounds #1 [5-Hydroxy-6,8-dimethoxy-2-oxo-2*H*-chromen-7-yl β-d-glucopyranoside], #16 [quercetagetin 7-(6″-(*E*)-caffeoylglucoside]), #18 [isopyrenin 7-*O*-glucoside], and #19 [12-hydroxy,*O*-[3,4,5-trihydroxybenzoyl-(06)-β-d-glucopyranoside] reduced their production in presence of one or both fungi. On the other hand, compounds #10 [3,4′,5,7-tetrahydroxyflavanone 3,7-Di-*O*-β-d-glucopyranoside], #14 [(*Z*)-resveratrol 3,4′-diglucoside], #20 [luteone 4,7-*O*-diglucoside], and #24 [(*R*)-1-*O*-[β-d-glucopyranosyl-(1-6)-β-d-glucopyranoside]-1,3-octanediol] showed an increased production in the metabolome of beans treated with *Trichoderma* alone or in combination with the pathogen. 

(v) Other polyphenols. Among the differentially accumulated metabolites, homologies were also found with isoflavanoids [#28: 2′-*O*-methylphaseollinisoflavan], isoflavanones [#25: 2′,4′,5,7-Tetrahydroxy-3′,8-diprenylisoflavanone], and isoflavans [#6: phaseollinisoflavan], which all decreased their production in presence of *T. velutinum* (CT028 and RT028), except for compound #6, whose production was DOWN-produced only in the three-way interaction (RT028) (Table 2). 

(vi) Phenols. Most of the phenolic compounds differentially produced by beans reduced their level in the presence of the beneficial fungus. This decrease was observed for compound #5 [homologous to hydroxytyrosol 1-O-glucoside], #31 [garcimangosone D], and #33 [gambogic acid], while compound #13 [similar to 1-*O*-sinapoylglucose] accumulated in higher amounts when T028 was present (CT028 and RT028), compared to the water-treated plant (Table 2).

### 2.7. Terpenes

The presence of *T. velutinum* and *R. solani* also affected the production of terpenes in bean leaves (Table 2). Thus, both dihydrophaseic acid 4-*O*-β-d-glucoside [#9, *m/z* 443.1923, RT 8.88 min, C_21_H_32_O_10_] and teucrein [#23, *m/z* 267.1235, at 17.52 min, C_16_H_12_O_4_], were UP-produced when *Trichoderma* was present alone, compared to the control. On the other hand, the plant metabolome showed an increased production of ethyl 7-epi-12-hydroxyjasmonate glucoside [#17, *m/z* 415.1971, RT 14.78 min, C_20_H_32_O_9_] in all the interactions examined, while an opposite trend was observed for compound #32, found to be similar to akeboside Ste [*m/z* 895.5050, RT 24.97 min, C_47_H_76_O_16_].

### 2.8. Principal Component Analysis (PCA) 

**Figure 4:**
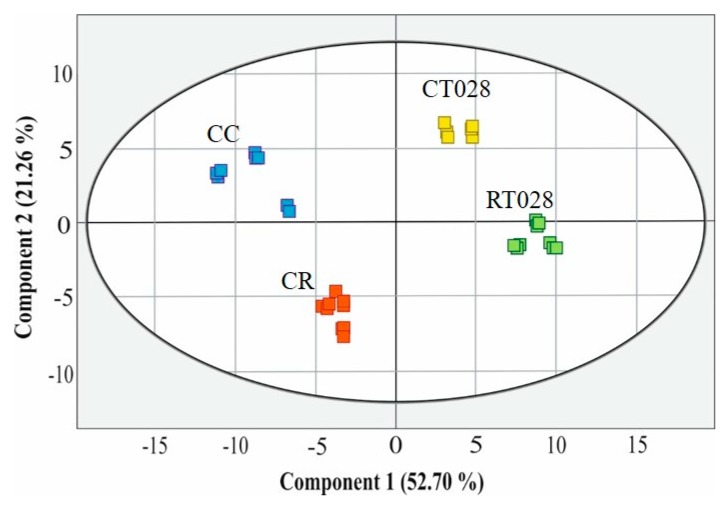
Principal components analysis of 36 compounds extracted from bean leaves subjected to different treatments. (Blue) control beans without pathogen and biocontrol agent (CC); (Red) beans that were infected with *R. solani* (CR); (Yellow) beans inoculated with *T. velutinum* (CT028); (Green) beans inoculated with *R. solani* and *T. velutinum* (RT028).

PCA was used to determine the relationship between the experimental treatments and the accumulation of selected phytochemical compounds in bean leaf metabolome. As shown in Figure 4, the first two components accounted for 73.96% of the total variance. Each metabolomic interaction was clustered separately. In particular, the plants infected with *R. solani* (CR) lie much closer to the control plants (CC), while the presence of *Trichoderma*, both alone (CT028) and together with the pathogen (RT028), also form a loose cluster (Figure 4).

## 3. Discussion

In this work, *T. velutinum* T028 was selected because of its positive effects on bean growth. Plants inoculated with this fungus showed significant increases in dry weights of both aerial parts and root system, also when the fungal pathogen *R. solani* was added to the substrate. Furthermore, both *T. velutinum* T028 and *R. solani* R43 isolates induced the expression of defense-related genes [8].

Plant-pathogen interactions are complex phenomena and affect the metabolism of each partner differently. Plant defense strategies against their pathogens are diverse, including the induction of expression of defense genes and also the production of antifungal compounds called phytoalexins, that include flavonoids, phenols, glucosinolates, terpenes and alkaloids with a broad spectrum of antimicrobial functions [27,28]. In this work, we have investigated the changes in metabolite profiles of bean plants cultivated for forty-five days in the presence of *T. velutinum* and/or *R. solani*. The metabolomic analysis of leaf extracts allowed the characterization of samples from different interaction conditions (plant alone, plant + pathogen, plant + antagonist, plant + pathogen + antagonist). Putative identification of the differentially accumulated metabolites in two- and three-way interactions, compared to control, revealed the increase or decrease in the accumulation of different classes of compounds, including polyphenols, terpenes, amino acids, carbohydrates, fatty acids, lipids, glycosides and peptides.

Many polyphenolic compounds with antibiotic activity act as phytoalexins in plant tissues during their interaction with phytopathogenic agents [29]. Phytoalexins are considered molecular markers of disease resistance [30] since they are synthesized *de novo* in response to pathogen attack [31]. Several biotic and abiotic factors induced the production of phytoalexins in different plant species of the Solanaceae, Leguminosae and Gramineae families [32]. Inoculation with non-pathogenic microorganisms, such as *Trichoderma* spp., or with hypovirulent pathogen strains induces systemic resistance in plant, resulting in the accumulation of phytoalexins at the site of infection and the production of antibiotic substances [2,33] e.g., accumulation of camalexin, a phytoalexin in *Arabidopsis,* in response to the presence of *Trichoderma*, which would indicate a further level of plant protection by these fungal strains [27,34]. 

Polyphenols comprise a large number of compounds, like phenols, phenolic acids, and flavonoids [11]. In this work, we found differences in the accumulation of flavonoids in plant leaves depending on the interaction condition (Table 2). Up to 8 different flavonoids (#1, 18, 19, 22, 24, 25, 26, 27) always resulted less abundant when plants were cultivated in presence of one or both fungi, compared to the non-inoculated control. Conversely, the production of 6 other flavonoids (#3, 10, 12, 14, 20, 21) was significantly increased whenever *Trichoderma* was present, but not in the sole presence or *R. solani*. Flavonoids protect plants against various biotic and abiotic stresses and exhibit a diverse spectrum of biological functions, playing an important role in the interaction between the plant and the environment [35]. The differences in the pattern of flavonoid accumulation observed in the metabolome of bean leaves could reflect the different metabolic pathways induced by the beneficial fungus rather than by the pathogenic one.

It is unclear whether the accumulation of different but structurally related phytoalexins in the plant (e.g., phaseollin, phaseollidin in the bean) is important for the resistance against pathogens [36]. In a previous study, when bean plants were inoculated with *R. solani,* a considerable amount of phaseollin accumulated in the lesions within 36 h after the treatment and was detected up to 12 days later [25]. We found a decreased production of two phytoalexin related compounds [#28: 2′-*O*-methylphaseollinisoflavan and #6: phaseollinisoflavan] in plants treated with *T. velutinum*.

The plant hormone jasmonic acid (JA) and its derivatives have been recognized as key regulators of defense responses against both biotrophic and necrotrophic pathogens [33]. We observed an over-accumulation of the terpene ethyl 7-epi-12-hydroxyjasmonate glucoside (#17; Table 2) in the presence of *T. velutinum* and/or *R. solani*, in comparison to control plants. This compound has been related to the metabolic pathway of JA, and its production was found to be induced when plants detect a microorganism. In this sense, Yogendra et al. [37] demonstrated that this terpene was detected when potato plants were in contact with the pathogen *Phytophthora infestans.* Similarly, we can hypothesize that both *Trichoderma* and *Rhizoctonia* activate the plant signaling cascade induced by this compound. However, other terpenes detected in the bean leaf metabolome showed a different accumulation pattern according to the different plant-microbe interactions analyzed in the present work (Table 2). Thus, compounds #9 (Dihydrophaseic acid 4-*O*-β-d-glucoside) and #23 (Teucrein) increased their accumulation only in presence of the beneficial fungus, while compound #32 (akeboside Ste) was less abundant in all the treated plants compared to controls. These evidences contribute to support that the presence of different beneficial of detrimental microbes in the rhizosphere may induce different plant metabolic pathways. Similar results were also observed with fatty acids and lipids, like γ-Linolenic acid (#35) and its derivative 13(S)-HOTrE (#34), both of which were produced at lower levels in the presence of the pathogen, compared to control plants. γ-Linolenic acid is the main precursor of jasmonate [38], which is responsible for the induction of a signalling cascade in response to non-pathogenic microorganisms [39]. In this work *R. solani* causes a reduction in the level of production of these compounds, which would result in a reduction in the ISR response, thereby facilitating plant invasion by the plant pathogen.

Bean plant metabolome also showed differences in peptide and amino acid accumulation. The presence of *T. velutinum* alone stimulated the production of l-Tryptophan (#8, Table 2), while the simultaneous occurrence of both microbes (RT028 treatment) stimulated the accumulation of its acetylated form (#15, *N*-acetyltryptophan). Conversely, *R. solani*-infected plants showed decreased amounts of both metabolites, compared to control. l-tryptophan has been found to stimulate the synthesis of auxins in the rhizosphere and trigger plant growth. The enhancement of plant biomass by promoting the growth of lateral root has been observed in many plant species treated with *Trichoderma* spp. and this effect has also been related to the production of indole-3-acetic acid (IAA) or auxin analogues [40,41]. In a similar way, the inoculation of *Vigna mungo* with other biocontrol agents (*Rhizobium* spp. and *Bacillus* spp.) increased yield up to 23.36% in presence of this compound [42].

Antifungal peptides derived from common bean demonstrated inhibitory activity against numerous plant pathogens, such as *Mycosphaerella arachidicola*, *R. solani*, *Verticillium dahliae, Setosphaeria turcica* and *Fusarium oxysporum* [43]. Antifungal activities of common bean peptides include modifications of hyphae morphology, membrane cell disruption, altered membrane permeabilization and induction of chitin accumulation at hyphal tips [43]. In our work, the accumulation of three peptides (Table 2) showed significant differences in plant leaves in presence of *T. velutinum* and/or *R. solani*. In particular, compounds #11 and #29 (homologs to the tripeptides Ile-Gln-Ile and Gln-Gln-Gln, respectively) were less abundant in bean plants treated with *Trichoderma* or *Rhizoctonia* than in controls. Interestingly, the dipeptide Valyl-Leucine (similar to compound #7) significantly increased after *T. velutinum* inoculation, and also in the presence of the pathogen. This could represent an example of metabolite production induced by the antagonistic interaction between *Trichoderma* and *R. solani*, and involved in the activation of plant defense responses.

## 4. Material and Methods

### 4.1. Fungal Isolates and Culture Conditions

The present study was conducted using strain *T. velutinum* T028, previously isolated from the Protected Geographical Indication (PGI) called “Alubia La Bañeza—León” (EC Reg. n.256/2010 published on 26 March 2010, OJEU L880/17). 

*R. solani* R43 is a highly virulent strain isolated from plants of the same PGI. This strain was stored in the collection “Pathogens and Antagonists of the Laboratory Diagnosis of Pests and Diseases” (PALDPD) at the University of Leon, Spain. 

The fungal isolates were grown on potato-dextrose-agar (PDA, Sigma-Aldrich, St. Louis, MO, USA) in the dark at 25 °C for one week. *Trichoderma* cultures were then exposed to light for 3–4 days in order to induce spore formation. Spores were collected and maintained at −80 °C in 50% glycerol suspensions.

### 4.2. Plant Material and Growth Conditions

Bean (*P. vulgaris* L. cv. Canela) seeds were germinated and cultured in four different conditions: (1) in presence of both *T. velutinum* (T028) and *R. solani* (R43) [RT028]; (2) T028 alone (CT028); (3) *R. solani* alone (CR); and (4) control without fungi (CC) (Figure 1).

Plants were grown in a climatic chamber; thirty pots (1 L capacity) with substrate, bentonite and cornmeal (100:5:2) were used per treatment, with two seeds per pot which were irrigated with 250 mL of water prior inoculation. Biomass of *R. solani* R43 was scraped from fungal cultures grown on PDA plates (5 plates per liter), homogenized and inoculated into the soil (50 mL per pot). For the control treatment, an uninoculated PDA medium was used. Pots were kept in a growth chamber for 8 days at 25 °C (16 h) and 16 °C (8 h), with 60% relative humidity in the dark. 

Bean seeds were surface sterilized (sodium hypochlorite 1% for 3 min and then rinsed with sterile distilled water for 6 min) and coated with a spore suspension (concentration 2 × 10^7^ spores mL^−1^ in 20 mL water for every 45 seeds) of *T. velutinum* (T028). Coated seeds were sown 8 days after inoculating the soil with the pathogen (Figure 1). Plants were grown in a climatic chamber under the conditions previously described [44], using a photoperiod of 16 h light/8 h dark, 25 °C/16 °C (day/night), 60% relative humidity and brightness of 3500 lux. The plants were harvested 45 days after sowing and their leaves were collected and lyophilized. In particular, three biological samples (obtained randomly from different pots within the same treatment) were processed per treatment.

### 4.3. Preparation of Bean Leaf Extracts

Extracts of plant leaves were obtained as described by Talhaoui et al. [45], with some modifications. Freeze-dried leaves (250 mg) were ground to a powder and extracted twice via Ultra-Turrax IKA T18 basic, using 10 mL of MeOH/H_2_O (80/20 *v*/*v*). Afterwards, samples were placed in an ultrasonic liquid processor (Sonics & Materials, Inc., Newtown, CT, USA) for 10 min, and centrifuged at 4000 rpm for 10 min at 4 °C. After solvent evaporation, extracts were reconstituted with 4 mL of MeOH:H_2_O (50:50 *v*/*v*) and filtered through a 0.2 µm membrane (Minisart^®^, Sartorius Stedim Biotech CA, Goettingen, Germany). 

### 4.4. LC-MS qTOF Conditions

Bean leaf extracts were separated on an Agilent HP 1260 Infinity Series liquid chromatograph coupled to a Q-TOF mass spectrometer, model G6540B (Agilent Technologies, Santa Clara, CA, USA). The chromatographic separations were performed using a Zorbax Eclipse Plus C_18_ analytical column (4.6 × 100 mm, 3.5 µm particle size) (Agilent Technologies, Santa Clara, CA, USA) as previously described [11] with slight modifications.

The mobile phases were treated with acidified water [H_2_O plus 0.1% formic acid (FA)] (eluent A) and acetonitrile (ACN) (ACN plus 0.1% FA) (eluent B). The chromatographic method consisted in the following linear gradient with a flow rate of 0.80 mL/min: 0 min, 0% B; 10 min, 20% B; 15 min, 30% B; 20 min, 50% B; 25 min, 75% B; 30 min, 100% B; 35 min, 100% B; 35.10 min, 0% B; and finally a 5 min post-run was used after each analysis. The injection volume was 5.0 µL and the column temperature was maintained at 25 °C.

The mass spectrometer was equipped with the model G6540B Dual ESI (Agilent Technologies, Santa Clara, CA, USA) source operating in negative ion mode. The optimum values of source parameters were: drying-gas temperature, 300 °C; drying-flow, 9 L/min; and nebulising-gas pressure, 45 psig. Two reference mass compounds were used to perform the real-time lock mass correction: purine (C_5_H_4_N_4_ at *m/z* 121.050873, 10 µmol L^−1^) and hexakis (1*H*,1*H*,3*H*-tetrafluoropentoxy)-phosphazene (C_18_H_18_O_6_N_3_P_3_F_24_ at *m/z* 922.009798, 2 µmol L^−1^). The capillary was maintained at 4000 V, fragmentor voltage at 180 V, cone 1 (skimmer 1) at 45 V, Oct RFV at 750 V. The spectra were recorded in the targeted mode as centroid spectra, with 3 scans per second, within the *m/z* mass range of 50–1100 amu. Each sample was run in triplicate, with 3 biological replicates per interaction examined. 

The MS data were processed through Agilent MassHunter Qualitative Analysis B.06.00 software (Agilent Technologies, Santa Clara, CA, USA), which provides a list of possible elemental formulas by using a molecular formula editor tool (ID Browser program, Agilent MassHunter Mass Profiler Software, Santa Clara, CA, USA). Sample normalization was performed as reported previously [16] by calculating the relative ratio of abundance of metabolites to all other peaks. According to the literature, the phytochemical compounds were tentatively identified based on the accurate mass measurements of the pseudomolecular ion [M-H]^−^, and comparing them to different databases containing more than 15.000 natural secondary metabolites (METLINE from Agilent Technologies; PubChem: https://pubchem.ncbi.nlm.nih.gov (access on 18 July 2018); ChemSpider: http://www.chemspider.com (access on 20 June 2018); HMDB: http://www.hmdb.ca (access on 30 May 2018); CAS: https://www.cas.org/cas-home (access on 15 April 2018); in-house plant metabolite database), as well as literature on *Fabaceae* species.

Statistical analyses were carried out using the Mass Profiler Professional Software 13.0 (G3835AA, Agilent Technologies, Santa Clara, CA, USA). Significant statistical differences among treatments (*p* < 0.05) were assessed by one-way ANOVA and principal component analysis (PCA). Leaf extracts obtained by each treatment were run in triplicate.

## 5. Conclusions

The metabolic profiles of bean leaves (*P. vulgaris*) were investigated by LC-MS analysis. As a result, 216 compounds were detected in the metabolome of bean leaves; among these, 36 metabolites accumulated differently in leaves inoculated with *T. velutinum* and/or *R. solani* compared to controls. Such metabolites were tentatively identified and included flavonoids, terpenes, carbohydrates, phenols, amino acids and peptides. *T. velutinum* treatments increased the accumulation of L- Tryptophan and its acetylated derivative in bean leaf metabolome, which could stimulate the synthesis of auxins in the rhizosphere and thereby stimulate plant growth. A derivative of JA, Ethyl 7-epi-12-hydroxyjasmonate glucoside, was found to increase in presence of *T. velutinum* and/or *R. solani* compared to control, thus suggesting the induction of a plant defense response effected by both fungi. However, a precursor of jasmonate (γ-Linolenic acid) found less accumulation in plants upon pathogen challenge, as compared to control. To the best of our knowledge, this work is the first report that investigates the effect of a biocontrol agent and/or a fungal pathogen on the metabolome of bean plants using an LC-MS-based analysis.

## Figures and Tables

**Table 1:** Phytochemical compounds determined in bean leaves by LC-MS analysis, including experimental mass, *m/z*, score, retention time (RT), formula, their putative identity and related references. Data refer only to compounds that accumulated differentially in leaves of plants inoculated with the fungal isolates compared to controls.

Peak	Experimental Mass	*m/z*	Score	Diff (ppm)	RT (min)	Formula	Putative Identification	Reference
**Amino acids**								
8	204.0898	203.0826 [M − H]^−^ 407.1733 [2M − H]^−^	49.94	−0.09	8.52	C_11_H_12_N_2_O_2_	l-Tryptophan	PubChem ID number—6305 [17]
15	246.1004	245.09315 [M − H]^−^	99.91	0.05	14.20	C_13_H_14_N_2_O_3_	*N*-acetyltryptophan	PubChem ID number—2002 [17]
**Peptides**								
7	230.1629	229.1557 [M − H]^−^	87.05	0.28	8.42	C_11_H_22_N_2_O_3_	Valyl-Leucine	PubChem ID number—107487
11	372.2371	371.2298 [M − H]^−^ 743.4673 [2M − H]^−^	99.84	0.41	10.00	C_17_H_32_N_4_O_5_	Ile Gln Ile	NA
29	402.1863	401.1788 [M − H]^−^	98.04	0.70	19.94	C_15_H_26_N_6_O_7_	Gln Gln Gln	NA
**Carbohydrates**								
2	342.1159	341.1087 [M − H]^−^	99.71	0.83	1.43	C_12_H_22_O_11_	Sucrose	PubChem ID number—5988 [17]
**Glycosides**								
4	338.0848	337.0775 [M − H]^−^	85.70	0.87	4.96	C_12_H_18_O_11_	l-Ascorbic acid-2-glucoside	PubChem ID number—54693473
**Fatty acids**								
35	278.22338	277.2169 [M − H]^−^ 555.4410 [2M − H]^−^	84.78	2.26	30.59	C_18_H_30_O_2_	γ-Linolenic acid	PubChem ID number—5280933 [17,18,19]
**Lipids**								
	Fatty acyls—Octadecanoids							
34	294.2195	293.2122 [M − H]^−^	99.67	0.76	26.29	C_18_H_30_O_3_	13(S)-HOTrE	PubChem ID number—47205624
	Glycerophosphates							
36	578.4296	577.4226 [M − H]^−^	90.87	0.43	30.59	C_31_H_63_O_7_P	PA(O-16:0/12:0)	PubChem number—52929565
	Fatty acyls glycoside							
30	334.1990	333.1916 [M − H]^−^	89.56	0.38	19.94	C_16_H_30_O_7_	3-*O*-α-l-rhamnopyranosyl-3-hydroxydecanoic acid	PubChem ID number—56936287 [20,21]
**Flavonoids**								
	Flavone							
22	374.0998	373.0926 [M − H]^−^	99.35	0.86	16.90	C_19_H_18_O_8_	3′,5-Dihydroxy-3,4′,6,7-tetramethoxyflavone	[21]
27	390.0951	389.0874 [M − H]^−^	82.88	1.10	18.32	C_19_H_18_O_9_	5,2′,4′-Trihydroxy-3,7,8,5′-tetramethoxyflavone	PubChem ID number—85296959
	Flavonol							
12	596.1739	595.1666 [M − H]^−^	99.67	0.33	10.27	C_26_H_28_O_16_	Quercetin 3-vicianoside	PubChem Id number—44259139 [11]
26	454.2409	453.2338 [M − H]^−^	99.76	0.29	18.05	C_20_H_38_O_11_	*n*-Octyl-β-d-maltopyranoside	NA
	Flavonol							
21	414.2248	413.2177 [M − H]^−^	82.59	0.37	16.25	C_29_H_50_O	Sitosterol	PubChem ID number—86821 [17,21]
	Flavonone							
3	580.1847	579.1773 [M − H]^−^	99.42	0.72	1.47	C_27_H_32_O_14_	Naringin	PubChem ID number—442428 [17]
	Flavonoids glycoconjugate							
1	400.1006	399.0930 [M − H]^−^	49.90	0.30	1.31	C_17_H_20_O_11_	5-Hydroxy-6,8-dimethoxy-2-oxo-2*H*-chromen-7-yl β-d-glucopyranoside	NA
10	612.1686	611.1613 [M − H]^−^	99.03	0.75	9.37	C_27_H_32_O_16_	3,4′,5,7-Tetrahydroxyflavanone 3,7-Di-*O*-β-d-glucopyranoside	CAS Number—80212-10-8 [21]
14	552.1841	551.1762 [M − H]^−^	97.55	1.60	11.13	C_26_H_32_O_13_	(*Z*)-Resveratrol 3,4′-diglucoside	PubChem ID number—22298557 [20]
16	642.1193	641.1119 [M − H]^−^	99.69	0.09	14.34	C_30_H_26_O_16_	Quercetagetin 7-(6″-(*E*)-caffeoylglucoside)	PubChem Id number—44259848
18	654.1769	653.1697 [M − H]^−^	99.89	−0.20	16.11	C_29_H_34_O_17_	Isopyrenin 7-*O*-glucoside	CAS Number—61252-86-6 [21]
19	540.1838	539.1766 [M − H]^−^	99.49	0.89	16.11	C_25_H_32_O_13_	12-Hydroxy, *O*-[3,4,5-trihydroxybenzoyl-(06)-β-d-glucopyranoside]	[20]
20	678.2894	677.2822 [M − H]^−^	85.36	0.98	16.24	C_32_H_39_O_16_	Luteone 4,7-*O*-diglucoside	[22,23]
24	470.2359	469.2287 [M − H]^−^	98.56	0.25	17.61	C_20_H_38_O_12_	(*R*)-1-*O*-[β-d-Glucopyranosyl-(1-6)-β-d-glucopyranoside]-1,3-octanediol	HMDB Id number—32799
	Isoflavanoid							
28	338.1522	337.1451[M − H]^−^ 319.1298 [M − H_2_O]^−^ 168.0663 [M − 2H]^−^	78.85	0.31	8.38	C_21_H_22_O_4_	2′-*O*-Methylphaseollinisoflavan	CAS Number—49594-01-6 [19,23,24]
	Isoflavanone							
25	424.2304	423.2234 [M − H]^−^	98.58	0.58	17.62	C_25_H_28_O_6_	2′,4′,5,7-Tetrahydroxy-3′,8-diprenylisoflavanone	CAS Number—64280-18-8 [24]
	Isoflavans							
6	324.1419	323.1346 [M − H]^−^ 647.2765 [2M − H]^−^	99.89	0.29	8.13	C_20_H_20_O_4_	Phaseollinisoflavan	PubChem ID number—4484952 [25]
**Phenols**								
	Tyrosols							
5	316.1153	315.1082 [M − H]^−^	97.00	1.57	7.29	C_14_H_20_O_8_	Hydroxytyrosol 1-*O*-glucoside	PubChem ID number—13845930
	Phenylpropanoids							
13	386.1201	385.1127 [M − H]^−^	94.90	3.17	10.71	C_17_H_22_O_10_	1-*O*-Sinapoylglucose	PubChem ID number—5280406
	Xanthonoid							
31	392.1102	391.1032 [M − H]^−^	97.71	1.07	21.10	C_19_H_20_O_9_	Garcimangosone D	PubChem ID number—11003703 [20]
33	628.3063	627.2991 [M − H]^−^	99.44	0.83	25.77	C_38_H_44_O_8_	Gambogic acid	PubChem ID number—5281632
**Terpenes**								
	Terpenoid							
9	444.1995	443.1923 [M − H]^−^	99.84	0.06	8.88	C_21_H_32_O_10_	Dihydrophaseic acid 4-*O*-β-d-glucoside	ChEBI Id number—23758 [26]
	Triterpenoid							
32	896.5127	895.5050 [M − H]^−^	98.64	0.47	24.97	C_47_H_76_O_16_	Akeboside Ste	PubChem ID number—46173935
	Terpene glycosides							
17	416.2046	415.1971 [M − H]^−^	99.53	0.52	14.78	C_20_H_32_O_9_	Ethyl 7-epi-12-hydroxyjasmonate glucoside	HMDB Id number—36340 [17]
	Terpene							
23	268.1308	267.1235 [M − H]^−^ 535.2548 [2M − H]^−^	49.76	1.09	17.52	C_14_H_20_O_5_	Teucrein	ChemSpider ID number—28944862

RT: retention time; NA: not available; CAS: https://www.cas.org/cas-home (access on 15 April 2018); ChemSpider: http://www.chemspider.com (access on 20 June 2018); HMDB: http://www.hmdb.ca (access on 30 May 2018); PubChem: https://pubchem.ncbi.nlm.nih.gov (access on 18 July 2018).

**Table 2:** Homologies of putatively identified compounds in the bean metabolome and changes in their accumulation pattern occurring when different interaction conditions (three-way, two-way and no interaction) were compared.

Id	Formula	Putative Identification	CT028 vs. CC ^a^	CR vs. CC ^a^	RT028 vs. CC ^a^
**Amino acids**					
8	C_11_H_12_N_2_O_2_	l-Tryptophan	Up	Down	--
15	C_13_H_14_N_2_O_3_	*N*-acetyltryptophan	--	Down	Up
**Peptides**					
7	C_11_H_22_N_2_O_3_	Valyl-Leucine	Up	Down	Up
11	C_17_H_32_N_4_O_5_	Ile Gln Ile	--	Down	Down
29	C_15_H_26_N_6_O_7_	Gln Gln Gln	Down	--	Down
**Carbohydrates**					
2	C_12_H_22_O_11_	Sucrose	Up	Up	--
**Glycosides**					
4	C_12_H_18_O_11_	l-Ascorbic acid-2-glucoside	--	Up	--
**Fatty acids**					
35	C_18_H_30_O_2_	γ-Linolenic acid	--	Down	Down
**Lipids**					
30	C_16_H_30_O_7_	3-*O*-α-l-rhamnopyranosyl-3-hydroxydecanoic acid	Down	--	Down
34	C_18_H_30_O_3_	13(S)-HOTrE	--	Down	--
36	C_31_H_63_O_7_P	PA(O-16:0/12:0)	--	Down	Down
**Flavonoids**					
1	C_17_H_20_O_11_	5-Hydroxy-6,8-dimethoxy-2-oxo-2*H*-chromen-7-yl β-d-glucopyranoside	Down	Down	Down
3	C_27_H_32_O_14_	Naringin	Up	--	--
6	C_20_H_20_O_4_	Phaseollinisoflavan	--	--	Down
10	C_27_H_32_O_16_	3,4’,5,7-Tetrahydroxyflavanone 3,7-Di-*O*-β-d-glucopyranoside	Up	--	Up
12	C_26_H_28_O_16_	Quercetin 3-vicianoside	--	--	Up
14	C_26_H_32_O_13_	(*Z*)-Resveratrol 3,4′-diglucoside	--	--	Up
16	C_30_H_26_O_16_	Quercetagetin 7-(6″-(*E*)-caffeoylglucoside)	--	Down	--
18	C_29_H_34_O_17_	Isopyrenin 7-*O*-glucoside	Down	Down	Down
19	C_25_H_32_O_13_	12-Hydroxy, *O*-[3,4,5-trihydroxybenzoyl-(06)-β-d-glucopyranoside]	Down	Down	Down
20	C_32_H_39_O_16_	Luteone 4,7-*O*-diglucoside	Up	Down	Up
21	C_29_H_50_O	Sitosterol	--	--	Up
22	C_19_H_18_O_8_	3′,5-Dihydroxy-3,4′,6,7-tetramethoxyflavone	Down	Down	Down
24	C_20_H_38_O_12_	(R)-1-*O*-[β-d-Glucopyranosyl-(1-6)-β-d-glucopyranoside]-1,3-octanediol	Down	--	Down
25	C_25_H_28_O_6_	2′,4′,5,7-Tetrahydroxy-3′,8-diprenylisoflavanone	Down	--	Down
26	C_20_H_38_O_11_	*n*-Octyl-β-d-maltopyranoside	Down	--	--
27	C_19_ H_18_ O_9_	5,2’,4′-Trihydroxy-3,7,8,5′-tetramethoxyflavone	Down	Down	Down
28	C_21_H_22_O_4_	2′-*O*-Methylphaseollinisoflavan	Down	--	Down
**Phenols**					
5	C_14_H_20_O_8_	Hydroxytyrosol 1-*O*-glucoside	Down	--	--
13	C_17_H_22_O_10_	1-*O*-Sinapoylglucose	Up	--	Up
31	C_19_H_20_O_9_	Garcimangosone D	Down	Down	Down
33	C_38_H_44_O_8_	Gambogic acid	Down	--	Down
**Terpenes**					
9	C_21_H_32_O_10_	Dihydrophaseic acid 4-*O*-β-d-glucoside	Up	--	--
17	C_20_H_32_O_9_	Ethyl 7-epi-12-hydroxyjasmonate glucoside	Up	Up	Up
23	C_14_H_20_O_5_	Teucrein	Up	--	--
32	C_47_H_76_O_16_	Akeboside Ste	Down	Down	Down

^a^ CC: Plant alone; CR: Plant + *R. solani*; CT028: Plant + *T. velutinum* T028; RT028: Plant + *T. velutinum* T028 + *R. solani.*
